# The dark side of nighttime all-terrain vehicle use

**DOI:** 10.1186/s40621-021-00316-y

**Published:** 2021-09-13

**Authors:** Charles A. Jennissen, Nicholas R. Stange, AnnaMarie Fjeld, Gerene M. Denning

**Affiliations:** 1 Department of Emergency Medicine, Roy J. and Lucille A. Carver College of Medicine, University of Iowa, Iowa City, 52242 USA; 2 Department of Pediatrics, Roy J. and Lucille A. Carver College of Medicine, University of Iowa, Iowa City, 52242 USA; 3 Saint Louis University School of Medicine, Saint Louis University, 1402 South Grand Boulevard, St. Louis, MO 63104 USA

**Keywords:** All-terrain vehicles, Nighttime driving, Youth, Helmets, Rollover, Roadway, Motor vehicle collision, Traumatic injury, Adult, Alcohol

## Abstract

**Background:**

Driving at night is considered a risk factor for all-terrain vehicle (ATV) crashes and injuries but few studies have addressed this issue. Our objective was to compare daytime and nighttime ATV crashes to better understand the potential risk factors associated with riding at night.

**Methods:**

A retrospective study was conducted on Iowa ATV-related crashes and injuries from January 1, 2002 through December 31, 2019 using four statewide datasets: the Iowa Department of Transportation (2002–2019), the Iowa Department of Natural Resources (2002–2019), the Iowa State Trauma Registry (2002–2018) and Iowa newspaper clippings (2009–2019). A standardized coding system was developed, and matching records were identified using Link Plus®. Descriptive (frequencies) and bivariate (chi-square, Fisher's exact test) analyses were performed using VassarStats (Statistical Computation Website).

**Results:**

Among crash victims where light conditions were documented (2125/3752, 57%), about one-quarter (485/2125, 23%) were injured at night. Nighttime crash victims were less likely youth (14% vs. 30%, *p* < 0.0001), less likely to be wearing helmets (11% vs. 18%, *p* = 0.003), and less frequently involved in motor vehicle crashes (7% vs. 14%, *p* < 0.0001) as compared to daytime victims. Nighttime victims were also more likely to be passengers (22% vs. 15%, *p* = 0.002), to test positive for alcohol (44% vs. 13% in adults, *p* < 0.0001), and to be injured on a roadway (53% vs. 45%, *p* = 0.007) and on weekends (76% vs. 63%, *p* < 0.0001). Numerous differences between daytime and nighttime characteristics were observed for males, females, and adults, whereas most characteristics were similar for youth. The severity of injuries and proportion of fatalities were similar among daytime and nighttime crash victims.

**Conclusions:**

Nighttime crash victims, particularly adults, were characterized by more frequent risky behaviors like carrying passengers, roadway riding, alcohol use, and lack of helmets. Whereas the frequency of risky behaviors among youth was similar for daytime and nighttime crashes, these behaviors put children at potential risk for injury. Multi-factorial, targeted injury prevention strategies are needed, including improved vehicle design, education about the dangers of nighttime operation, and passage and enforcement of ATV safety laws. Particularly relevant to our study are laws that prohibit nighttime riding.

## Background

All-terrain vehicles (ATVs) continue to represent a significant public health and safety concern. Since 2011, annual ATV-related fatalities estimated by the Consumer Product Safety Commission (CPSC) in the U.S. were 651–743, and estimated injuries treated in emergency departments (ED) ranged from 81,800–107,900 per year (Topping 2018). Children continue to be a significant proportion of those injured (~one-third) and killed (~one-quarter) (Denning and Jennissen 2018). In fact, more children less than 16 years of age die from ATVs than from bicycle crashes (Helmkamp et al. 2009).

Children have a 12 times greater risk of injury while riding ATVs as compared to middle-aged adults (Rodgers and Adler 2001), and younger age is an independent risk factor for ATV crashes (Denning et al. 2014). Other universal risk factors for ATV-related crashes and injuries include roadway riding, lack of helmet use, speed, being male, lack of training, operating under the influence of drugs or alcohol, age-inappropriate vehicle size, lack of youth supervision, carrying passengers, and riding at night (Denning and Jennissen 2018; Aitken et al. 2004; Consumer Federation of America 2021).

Few studies have addressed nighttime ATV riding. Epidemiologic studies (including all ages) have reported nighttime ATV crashes accounted for 27–38% of victims (Lord et al. 2010; Rostas et al. 2013; Williams et al. 2014; Jennissen et al. 2016a). Additionally, a multicenter study of pediatric patients from three states reported that 16% of the crashes involving youth occurred at night (Mazotas et al. 2014). Finally, a study on intracranial hemorrhage in ATV crash victims found a positive association between alcohol intoxication and nighttime crashes (Rostas et al. 2013).

In addition to retrospective research, survey studies of youth at Connecticut agricultural fairs and of 4-H members in central Illinois found 46 and 53% had ridden ATVs after dark, respectively (Campbell et al. 2010; Hafner et al. 2010). In both studies, those who reported being injured or being in a crash had higher proportions of riding after dark. The objective of this study was to compare and contrast daytime and nighttime ATV crashes to better understand the potential risk factors associated with riding at night.

## Methods

### Study design

A retrospective study was conducted on ATV-related crashes and injuries that occurred in Iowa from January 1, 2002 through December 31, 2019 using our statewide off-road vehicle (ORV) injury surveillance database. Matching records from original data sources were identified using Link Plus®, available from the Centers for Disease Control and Prevention. The University of Iowa Institutional Review Board (IRB) approved this study.

### ORV injury surveillance database

The ORV injury surveillance database combines crash and injury records from four statewide sources: the Iowa Department of Transportation (DOT), the Iowa Department of Natural Resources (DNR), the Iowa State Trauma Registry (STR) and newspaper clippings (Denning et al. 2013a; Denning et al. 2013b; Jennissen et al. 2016b; Qin et al. 2017). Access to all data was in compliance with federal, state, and local regulations. Press clippings of ORV crashes were obtained through the media monitoring service, newzgroup^SM^ (Jennissen et al. 2016a).

Each dataset had unique fieldnames and coding systems, and except for DOT data, several variables required coding from narratives. Moreover, the DOT coding system was revised in 2015 and the STR transitioned from ICD-9 to ICD-10 coding systems in 2014. Therefore, a standardized coding system was developed similar to those previously described (Denning et al. 2013a; Denning et al. 2013b; Qin et al. 2017). Best practices were used for coding of database narratives and news reports, i.e., initial independent coding by two team members and resolution of coding discrepancies or questions regarding entries by team discussion, including senior research staff.

### Identifying ATV crashes

To identify ATV crashes for inclusion in this study, we used several strategies. For DOT data, combinations of DOT-assigned vehicle codes for off-road vehicles and where available, vehicle make, model, description, and manufacturer identification number (VIN) were used. For DNR data, the variable “vehicle type”, as well as the make, model, and crash narratives were used. For STR data, e-codes for off-road vehicle crashes were used to request initial data and then cause-of-injury narratives were used for further identification. Press clippings were requested from the service based on keyword searches for ATVs (e.g., ATV, 4-wheeler, 3-wheeler). Crashes involving side-by-side vehicles, including utility task vehicles and recreational off-highway vehicles (ROVs), were excluded. We identified matching records and created the merged dataset using the following sequence: all DOT cases, STR cases without DOT matches, DNR cases without DOT and STR matches, and newspaper clippings without DOT, STR, and DNR matches. Only victims who were riders on the vehicle (operator or passenger) at the time of the crash were included. Other victims, e.g., pedestrians, were excluded. The final N for analysis was 3752 cases: DOT, 797; DNR, 270; STR, 2541; Press Clippings, 144.

### Study variables

For analysis, we used variables that were moderately (e.g., crash mechanism) to well documented (demographics). Person-related variables used were the victim’s sex, age, seating position, helmet use, alcohol use (documented in DOT and STR datasets), whether the injury mechanism included falling or being ejected from the vehicle and/or whether it included being hit or pinned by the vehicle, and whether the injury was fatal. Youth were defined by convention as < 16 years based on the fact that adult-size ATVs are designed for those 16 years of age and older. Injury information was determined using the STR dataset and included the presence (GCS < 15) or absence (GCS = 15) of brain injuries based on the Glasgow Coma Scale (GCS). Injury severity was based on the Injury Severity Score (ISS) and was dichotomized to > 15 (major trauma) and ≤ 15 for comparative analysis (Boyd et al., 1987).

The number of wheels was the only vehicle-related variable utilized in the study. Crash-related variables included time (season, day of week), light conditions, location (e.g., roadway), roadway surface (paved vs. unpaved), and crash mechanism (e.g., rollover). Light conditions were coded as dawn (up to 30 min before sunrise), day, dusk (up to 30 min after sunset), and night. For bivariate comparisons (Day versus Night), we excluded Dawn and Dusk as each was too small in number to use independently in crosstab analysis. Moreover, neither consistently reflected the characteristics of either daytime or nighttime crashes, so grouping them with one or the other was not justified.

### Documentation of light conditions

Documentation of light conditions was high for the DOT (788/797, 99%), DNR (242/270, 90%), and press clippings (124/144, 86%). In contrast, overall documentation in the STR was 38% (971/2541). This was due to highly limited recording of the crash time in ICD-9 records (2002–2013), whereas documentation was 90% (735/818) after ICD-10 coding was implemented in 2014. Overall documentation was 57% (2125/3752).

### Data analysis

Descriptive analyses (frequencies) and comparative analyses (chi square test or Fisher’s exact probability test for cell sizes < 5) were performed using the Vassar Website for Statistical Calculations (http://vassarstats.net/). Missing data were not included in analysis. All *p* values were two-tailed and statistical significance was defined as *p* < 0.05.

## Results

### Crash victim characteristics

There was a total of 3752 ATV crash victims in the database. Females, youth < 16 years of age, and passengers constituted 22, 26, and 16% of victims, respectively (Table 1). Overall, one in five victims who were tested for alcohol were positive. The most common season for crash-related injuries was summer, constituting 41% of cases. Two-thirds of victims were injured on weekends, both Saturday (1007/3752) and Sunday (1005/3752) having 27% of cases each. Over one-third of injuries occurred on a roadway and more than two-fifths of these roadway-related injuries occurred on unpaved roads. The most common injury mechanism was a non-collision event (e.g., rollover) involving nearly three-fourths of those injured. Over two-thirds of all riders fell or were ejected from the vehicle and one-third were hit or pinned by the ATV. Four percent of cases were fatal. In data not shown, only 1% of crash victims (40/3752) were riding three-wheelers. Of the 2125 cases documented for light condition, 11 (1%) were at dawn, 1501 (71%) were during the day, 128 (6%) were at dusk, and 485 (23%) occurred at night.

**Table 1:** Overall characteristics of ATV crash victims and comparisons between victims from daytime and nighttime crashes as documented in the Iowa Statewide ORV Database

	All	Day	Night	
N (Row%)	3752	1501 (76%)	485 (24%)	
Variable	n (Col%)^a^	n (Col%)^a^	n (Col%)^a^	*p* value
Sex				
Male	2896 (78%)	1176 (79%)	371 (77%)	0.40
Female	817 (22%)	308 (21%)	108 (21%)	
Age Range				
Youth (< 16)	961 (26%)	444 (30%)	66 (14%)	< 0.0001
Adult	2716 (74%)	1020 (70%)	407 (86%)	
Seating				
Operator	2156 (78%)	1039 (85%)	302 (78%)	0.002
Passenger	421 (16%)	187 (15%)	85 (22%)	
Helmet				
Yes	413 (19%)	164 (18%)	32 (11%)	0.003
No	1722 (81%)	732 (82%)	263 (89%)	
Alcohol^b^				
Positive	356 (21%)	100 (10%)	135 (39%)	< 0.0001
Negative	1346 (79%)	857 (90%)	210 (61%)	
Not tested/unknown	1636	282	70	
Season				
Winter	352 (9%)	131 (9%)	57 (12%)	0.0012
Spring	1010 (27%)	399 (27%)	103 (21%)	
Summer	1526 (41%)	653 (44%)	191 (39%)	
Fall	864 (23%)	318 (21%)	134 (28%)	
Weekday				
Weekday (Mon-Thu)	1291 (34%)	555 (37%)	118 (24%)	< 0.0001
Weekend (Fri-Sat)	2461 (66%)	946 (63%)	367 (76%)	
Location				
Roadway	980 (35%)	554 (45%)	207 (53%)	0.007
Off-road	1815 (65%)	678 (55%)	185 (47%)	
Road surface				
Paved	324 (42%)	218 (45%)	79 (44%)	0.72
Unpaved	441 (58%)	264 (55%)	102 (56%)	
Crash mechanism				
ATV-ATV collision	132 (4%)	57 (4%)	33 (8%)	< 0.0001
ATV-MV collision	247 (8%)	186 (14%)	30 (7%)	
ATV-Object collision	461 (15%)	152 (12%)	70 (17%)	
Non-collision	2316 (73%)	896 (69%)	290 (69%)	
Fell/Ejected				
Yes	1086 (69%)	407 (62%)	137 (68%)	0.11
No	480 (31%)	254 (38%)	65 (32%)	
Hit/Pinned				
Yes	499 (33%)	194 (24%)	45 (17%)	0.024
No	1033 (67%)	628 (76%)	220 (83%)	
Fatality				
Yes	131 (4%)	69 (5%)	30 (6%)	0.16
No	3608 (96%)	1423 (95%)	451 (94%)	

*Abbreviations*: all-terrain vehicle, ATV; column percent, Col%; MV, motor vehicle; off-road vehicle, ORV

^a^Column total may not equal group N due to missing data

^b^Alcohol test results are from the Department of Transportation and State Trauma Registry datasets: All *N* = 3338; Day *N* = 1239; Night *N* = 415

### Daytime versus nighttime crashes

When comparing the characteristics of victims in crashes that occurred in daytime to those at night, a number of differences were observed (Table 1). Relative to daytime victims, nighttime victims had a lower proportion of youth < 16 years old (14% vs. 30%, *p* < 0.0001), and a higher proportion were passengers on the vehicle (22% vs. 15%, *p* = 0.002). Nighttime victims were more frequently un-helmeted (89% vs. 82%, *p* = 0.003). Among all riders who were tested, a higher proportion tested positive for alcohol when comparing nighttime to daytime crashes (39% vs. 10%, *p* < 0.0001). Nighttime crash victims were more likely to be injured in the fall (28% vs. 21%) and winter (12% vs. 9%), and less likely to be injured in the spring (21% vs. 27%) and summer (39% vs. 44%) when compared to daytime victims, overall *p* = 0.0012. A higher proportion of nighttime victims were observed in weekend crashes (76% vs. 63%, *p* < 0.0001) and on public roadways (53% vs. 45%, *p* = 0.007). The proportion of victims of ATV-ATV collisions was higher (8% vs. 4%) whereas the proportion injured in collisions with other motor vehicles was lower (7% vs. 14%) at night as compared to during the day, overall *p* < 0.0001. In addition, the proportion of victims hit or pinned by the vehicle at night was lower (17% vs. 24%, *p* = 0.024), as compared to during the day.

### Comparison of daytime versus nighttime crashes by sex

Approximately one-quarter of crashes with male (371/1547, 24%) and with female (108/416, 26%) victims occurred at night (Table 2). For both males (11% vs. 27%, *p* < 0.0001) and females (24% vs. 42%, *p* = 0.0014), a lower proportion of youth as compared to adults were injured in nighttime crashes than in daytime ones. Males were less frequently helmeted at night than during the day (12% vs. 20%, *p* = 0.009). The proportion of male (39% vs. 11%) and of female (40% vs. 7%) crash victims testing positive for alcohol was higher in nighttime versus daytime crashes, *p* < 0.0001 in each case. Crashes with male victims occurred more frequently at night than during the day in the fall (27% vs. 22%) and in the winter (14% vs. 10%), overall *p* = 0.016, as well as on weekends (73% vs. 62%, *p* < 0.0001). For females, the proportion injured in nighttime versus daytime crashes were also higher in the fall (31% vs. 18%, overall *p* = 0.029) and on weekends (83% vs. 66%, *p* = 0.0008). As compared to daytime, nighttime ATV-ATV collisions more frequently involved males (7% vs. 4%) and motor vehicle collisions less frequently did so (8% vs. 16%), overall *p* < 0.0001. In addition, lower proportions of males were hit or pinned by the vehicle (18% vs. 26%, *p* = 0.021) in nighttime as compared to daytime crashes.

**Table 2:** Comparisons by light conditions (day versus night) for male and female ATV crash victims as documented in the Iowa Statewide ORV Database

	Male			Female		
	Day	Night		Day	Night	
N (Row%)	1176 (76%)	371 (24%)		308 (74%)	108 (26%)	
Variable	n (Col%)^a^	n (Col%)^a^	*p* value	n (Col%)^a^	n (Col%)^a^	*p* value
Age Range						
Youth (< 16)	318 (27%)	40 (11%)	< 0.0001	124 (42%)	25 (24%)	0.0014
Adult	843 (73%)	327 (89%)		174 (58%)	79 (76%)	
Seating						
Operator	881 (91%)	266 (88%)	0.05	149 (60%)	35 (45%)	0.016
Passenger	84 (9%)	38 (13%)		98 (40%)	43 (55%)	
Helmet						
Yes	140 (20%)	29 (12%)	0.009	24 (12%)	3 (5%)	0.15
No	557 (80%)	204 (88%)		171 (88%)	58 (95%)	
Alcohol^c^						
Positive	85 (11%)	101 (39%)	< 0.0001	15 (7%)	34 (40%)	< 0.0001
Negative	665 (89%)	157 (61%)		187 (93%)	52 (60%)	
Not tested/unknown	221	57		60	13	
Season						
Winter	116 (10%)	52 (14%)	0.016	13 (4%)	5 (5%)	0.029
Spring	303 (26%)	79 (21%)		86 (28%)	22 (20%)	
Summer	496 (42%)	141 (38%)		153 (50%)	47 (44%)	
Fall	261 (22%)	99 (27%)		56 (18%)	34 (31%)	
Weekday						
Weekday (Mon-Thu)	447 (38%)	99 (27%)	< 0.0001	104 (34%)	18 (17%)	0.0008
Weekend (Fri-Sat)	729 (62%)	272 (73%)		204 (66%)	90 (83%)	
Location						
Roadway	424 (44%)	155 (52%)	0.02	127 (50%)	51 (58%)	0.19
Off-road	539 (56%)	145 (48%)		127 (50%)	37 (42%)	
Road surface						
Paved	176 (48%)	56 (42%)	0.24	41 (37%)	22 (48%)	0.20
Unpaved	193 (52%)	78 (58%)		70 (63%)	24 (52%)	
Crash mechanism						
ATV-ATV collision	38 (4%)	24 (7%)	< 0.0001	17 (6%)	9 (9%)	0.12
ATV-MV collision	156 (16%)	25 (8%)		28 (10%)	4 (4%)	
ATV-Object collision	121 (12%)	53 (17%)		30 (11%)	16 (16%)	
Non-collision	685 (69%)	219 (68%)		200 (73%)	68 (70%)	
Fell/Ejected						
Yes	314 (61%)	109 (69%)	0.058	89 (64%)	26 (60%)	0.63
No	200 (39%)	48 (31%)		49 (36%)	17 (40%)	
Hit/Pinned						
Yes	165 (26%)	36 (18%)	0.021	29 (17%)	9 (15%)	0.69
No	476 (74%)	166 (82%)		142 (83%)	52 (85%)	
Fatality						
Yes	63 (5%)	27 (7%)	0.16	6 (2%)	3 (3%)	0.70^b^
No	1108 (95%)	340 (93%)		302 (98%)	105 (97%)	

*Abbreviations*: all-terrain vehicle, ATV; column percent, Col%; MV, motor vehicle; off-road vehicle, ORV

^a^Column total may not equal group N due to missing data

^b^Fisher’s Exact Test

^c^Alcohol test results are from the Department of Transportation and State Trauma Registry datasets: Male/Day *N* = 971; Male/Night *N* = 315; Female/Day *N* = 262, Female/Night *N* = 99

### Comparison of daytime versus nighttime crashes by age

The characteristics of daytime and nighttime crashes were similar for youth victims with a few exceptions, whereas numerous differences were seen for adults injured at night versus during the day (Table 3). Relative to daytime, adults injured at night had a higher proportion of passengers (18% vs. 9%, *p* < 0.0001), and a lower proportion of helmeted riders (10% vs. 17%, *p* = 0.011). For adults, documented alcohol use was higher at night than during the day (44% vs. 13%, *p* < 0.0001). In the fall, nighttime crashes had higher proportions of both youth (35% vs. 20%, overall *p* = 0.021) and adult victims (27% vs. 22%, overall *p* = 0.046) than daytime ones. A higher proportion of adult victims was also seen on weekends (76% vs. 63%, *p* < 0.0001) when comparing daytime and nighttime crashes. Youth victims injured in crashes on paved roadways were more common at night then during the day (65% vs. 41%, *p* = 0.032). As compared to during the day, youth victims in nighttime crashes were also more frequently injured in ATV-ATV collisions (17% vs. 7%) and less frequently injured in ATV collisions with other motor vehicles (4% vs. 16%), overall *p* = 0.0042. At night as compared to day, adult victims were more commonly in ATV-ATV collisions (7% vs. 3%) and less commonly in crashes involving collisions with other vehicles (7% vs. 14%), overall *p* = 0.0002. Adult nighttime crashes less frequently involved victims being hit or pinned by the vehicle (17% vs. 26%, *p* = 0.008) relative to daytime crashes.

**Table 3:** Comparisons by light conditions (day versus night) for youth and adult ATV crash victims as documented in the Iowa Statewide ORV Database

	Youth			Adult		
	Day	Night		Day	Night	
N (Row%)	444 (87%)	66 (13%)		1020 (71%)	407 (29%)	
Variable	n (Col%)^a^	n (Col%)^a^	*p* value	n (Col%)^a^	n (Col%)^a^	*p* value
Sex						
Male	318 (72%)	40 (62%)	0.085	843 (83%)	327 (81%)	0.29
Female	124 (28%)	25 (38%)		174 (17%)	79 (19%)	
Seating						
Operator	256 (65%)	31 (65%)	0.29	760 (91%)	268 (82%)	< 0.0001
Passenger	100 (28%)	17 (35%)		78 (9%)	59 (18%)	
Helmet						
Yes	61 (21%)	7 (16%)	0.46	101 (17%)	25 (10%)	0.011
No	227 (79%)	36 (84%)		498 (83%)	224 (90%)	
Alcohol^b^						
Positive	6 (2%)	2 (5%)	0.60^c^	93 (13%)	133 (44%)	< 0.0001
Negative	242 (98%)	35 (95%)		609 (87%)	168 (56%)	
Not tested/Unknown	111	18		171	52	
Season						
Winter	27 (6%)	3 (5%)	0.021	101 (10%)	52 (13%)	0.046
Spring	121 (27%)	9 (14%)		267 (26%)	92 (23%)	
Summer	205 (46%)	31 (47%)		429 (42%)	154 (38%)	
Fall	91 (20%)	23 (35%)		223 (22%)	109 (27%)	
Weekday						
Weekday (Mon-Thu)	162 (36%)	18 (27%)	0.14	379 (37%)	99 (24%)	< 0.0001
Weekend (Fri-Sat)	282 (64%)	48 (73%)		641 (63%)	308 (76%)	
Location						
Roadway	159 (44%)	27 (56%)	0.10	386 (46%)	173 (52%)	0.074
Off-road	204 (56%)	21 (44%)		450 (54%)	160 (48%)	
Road surface						
Paved	53 (41%)	15 (65%)	0.032	163 (47%)	59 (39%)	0.11
Unpaved	76 (59%)	8 (35%)		186 (53%)	92 (61%)	
Crash mechanism						
ATV-ATV collision	25 (7%)	9 (17%)	0.0042	30 (3%)	24 (7%)	0.0002
ATV-MV collision	59 (16%)	2 (4%)		121 (14%)	26 (7%)	
ATV-Object collision	53 (14%)	12 (22%)		97 (11%)	56 (16%)	
Non-collision	241 (64%)	31 (57%)		632 (72%)	251 (70%)	
Fell/Ejected						
Yes	133 (65%)	24 (67%)	0.86	268 (60%)	108 (67%)	0.14
No	71 (35%)	12 (33%)		175 (40%)	53 (33%)	
Hit/Pinned						
Yes	42 (18%)	7 (19%)	0.82	146 (26%)	37 (17%)	0.008
No	193 (82%)	29 (81%)		425 (74%)	185 (83%)	
Fatality						
Yes	15 (3%)	0 (0%)	0.24^c^	54 (5%)	30 (7%)	0.13
No	429 (97%)	66 (100%)		962 (95%)	374 (93%)	

*Abbreviations*: all-terrain vehicle, ATV; column percent, Col%; MV, motor vehicle; off-road vehicle, ORV

^a^Column total may not equal group N due to missing data

^b^Alcohol test results are only from the Department of Transportation and State Trauma Registry datasets: Youth/Day *N* = 359; Youth/Night *N* = 55; Adult/Day *N* = 873, Adult/Night *N* = 353

^c^Fisher’s Exact test

### Injuries

Brain injuries and major trauma were observed in 10 and 14% of cases, respectively, using data in the trauma registry (Table 4). These injuries were not different by sex, but adults suffered major trauma more frequently than youth (16% vs. 10%, *p* = 0.0003). There were no observed differences in the frequency of brain injuries and of major versus minor trauma for those injured in daytime vs. nighttime crashes.

**Table 4:** Injuries from ATV crashes as documented in the Iowa Statewide ORV Database^a^

	All	Male	Female	*p* value	Youth	Adult		Day	Night	
N (Row%)	2937	2276 (78%)	659 (22%)		780 (27%)	2151 (73%)		778 (76%)	245 (24%)	
	n (Col%)^b^	n (Col%)^b^	n (Col%)^b^		n (Col%)^b^	n (Col%)^b^	*p value*	n (Col%)^b^	n (Col%)^b^	*p* value
Brain injury										
Yes	208 (10%)	165 (11%)	43 (10%)	0.65	50 (10%)	158 (11%)	0.43	74 (10%)	30 (13%)	0.25
No	1775 (90%)	1383 (89%)	391 (90%)		471 (90%)	1301 (89%)		664 (90%)	207 (87%)	
Trauma										
Minor	2388 (86%)	1847 (85%)	539 (87%)	0.18	662 (90%)	1723 (84%)	0.0003	659 (87%)	203 (85%)	0.35
Major	398 (14%)	320 (15%)	78 (13%)		76 (10%)	322 (16%)		99 (13%)	37 (15%)	

*Abbreviations*: all-terrain vehicle, ATV; column percent, Col%; off-road vehicle, ORV

^a^Data from the Iowa State Trauma Registry

^b^Column total may not equal N due to missing data

## Discussion

Although nighttime riding is considered a risk factor for ATV crash, relatively few studies have provided data on the nighttime use of ATVs and resulting injuries. We found that approximately one-fourth of ATV crashes occurred at night. Other studies have found similar proportions. About one-third of ATV-related fatalities documented in Ontario coroner reports from 1996 to 2005 (*N* = 74) occurred after dusk and before dawn (Lord et al. 2010). Of 481 ATV crash victims presenting to the University of Mississippi Medical Center from 2005 to 2010, 27% of the crashes occurred between 8 pm and 6 am (Rostas et al. 2013). A newspaper clippings study of off-road vehicle crashes in nine Great Plains states (*N* = 1019) found that 38% of ATV crashes had occurred in compromised light conditions (dusk, night, dawn) (Jennissen et al. 2016a). In addition, a study of U.S. roadway ATV fatalities utilizing the Fatality Analysis Reporting System (FARS) revealed 28% occurred from 9 pm-6 am (Williams et al. 2014).

### Demographics of nighttime crashes

About one-quarter of all males and females were injured in nighttime crashes. Although a West Virginia study reported males having a higher proportion of crashes at night (30%) as compared to females (14%), this difference was not statistically significant (Touma et al. 1999). With respect to age in our study, the proportion of nighttime crashes was significantly higher for adults (29%) than for youth (13%). Previous research on ROVs also found adults to have a higher percentage of crashes at night as compared to youth. Specifically, ~ 40% of adult ROV crash victims were injured at night as compared to 0% of youth victims treated at the University of Iowa and 17% of youth in press clippings of ROV crashes in nine Great Plains states, *p* = 0.004 (Jennissen et al. 2016a; Jennissen et al. 2016c). Our observed proportion of youth injured at night was also similar to the 16% of pediatric nighttime victims seen in a previous multi-institutional study (Mazotas et al. 2014).

Our study also showed that higher proportions of female and adult passenger victims were seen in nighttime as compared to daytime crashes. In contrast, there were no differences in the proportion of ATV passenger victims when comparing crashes occurring during the day and at night for ED patients evaluated at the University of Iowa (Jennissen et al. 2016b). This difference may reflect the broader sampling of crashes and injuries in our statewide database.

Helmet use was lower at night than during the day. Limited helmet use has been reported in numerous studies, including lower use by females as compared to males and adults versus youth (Denning et al. 2013c; Merrigan et al. 2011). This is the first study to report a difference in helmet use between daytime and nighttime crashes.

### Alcohol use

We found that adult victims in ATV crashes occurring at night more frequently tested positive for alcohol than victims in daytime crashes (44% vs 13% of those tested). A small number of youth victims tested positive, and the proportion positive was not different by day versus night. Other studies also found a significantly higher proportion of alcohol use among ATV crash victims at night as compared to during the day. At the University of Mississippi, nearly one-half (46%) of nighttime crash victims were alcohol intoxicated while about one-fifth (22%) were positive during daylight hours (Rostas et al. 2013). Similarly, 52% of nighttime ATV crashes were alcohol-related as compared to 19% during the day for ED patients seen at the University of West Virginia (Touma et al. 1999). Alcohol intoxication has been found to be an independent risk factor for serious ATV-related injury (Rostas et al. 2013), including maxillofacial (Touma et al. 1999) and spinal injuries (Sanfilippo et al. 2008).

### Seasonality and day of the week

Nighttime crash victims were more common in fall and winter as compared to daytime crash victims that were also of higher frequency in spring and summer. We speculate this may reflect similar evening riding habits combined with shorter days in the fall and winter. The most common nights for ATV-related injuries in our study were Saturday and Sunday. The University of West Virginia similarly found Saturday to be the most frequent day of the week for nighttime ATV crashes (Touma et al. 1999).

### Crash location

Overall, a higher proportion of roadway crash victims was seen at night and injuries occurred on both paved and unpaved surfaces. Previous studies showed that riding on the road is an independent risk factor for deaths and serious injury and that injury severity was higher for both paved and unpaved roads relative to off-road terrains (Denning et al. 2013a; Denning et al. 2013c; Denning and Jennissen 2016).

### Crash mechanism

Injuries from ATV-ATV collisions were more frequent at night, whereas those from collisions with other motor vehicles were less frequent. This was true for males, youth, and adult victims. We speculate that limited light conditions and visibility may contribute to the higher frequency of ATV-ATV collisions when riding in groups and that the lower frequency of collisions with other motor vehicles may reflect less traffic at night. As previously observed (Denning et al. 2014; Denning et al. 2013a; Qin et al. 2017; Denning et al. 2013c; Denning and Jennissen 2016), the major crash mechanism in all cases was a non-collision event, i.e., a rollover. A study of ATV-related spinal injuries noted rollovers to be a frequent injury mechanism of their nighttime patients (Sanfilippo et al. 2008).

### Injury mechanism and outcome

Two-thirds of nighttime and daytime crash-related injuries involved falling from or being ejected from the vehicle. Conversely, being hit or pinned by the vehicle was less frequently observed at night (17%) than during the day (26%). The basis for this difference remains to be determined. An earlier study reported that the percentage of crush-related injuries (e.g. compression asphyxia) in fatal ATV crashes had increased over time (Denning et al. 2013c). As ATVs have increased in size and weight, the ability of riders to self-eject or clear the vehicle during a mishap has decreased leading to a greater likelihood of being hit or pinned.

We did not find differences in brain injuries or trauma severity when comparing nighttime and daytime crash victims despite differences in alcohol use and injury mechanism. Additional studies may be needed, as documentation of light conditions was highly limited in the STR, the primary source of injury data. In contrast, a regression analysis of snowmobile-related deaths found fatalities to be about twice as likely during times of sub-optimal lighting as compared to daylight hours (Rowe et al. 1994).

### Prevention

Our study builds on previous knowledge in the field, particularly on the limited studies related to light conditions, and provides support for the recommendations that driving ATVs at night by adults should only be done with extreme caution and avoided if at all possible. Youth have been shown to be a particularly vulnerable ATV population and should never be riding ATVs at night.

Among strategies that could reduce deaths and injuries, including among riders who choose nighttime riding are improvements in ATV design (Jennissen et al. 2018). These would include brighter lights on adult-size vehicles (Lord et al. 2010). Moreover, because the vast majority of pediatric deaths and injuries occur with adult-size vehicles, design changes, like seat design, that discourage youth riding would be of value and could prevent riding of these vehicles both at night and during the day (Jennissen et al. 2014). Promising results have also been seen in studies of crush protection devices that prevent or reduce the severity of being hit/pinned by the ATV (Lower and Trotter 2014; Meyers 2016).

One of the most effective prevention strategies is passage and enforcement of safety laws. Whereas many states have ATV-related legislation to address safety concerns, state laws vary considerably (Specialty Vehicle Institute of America (SVIA) 2021), and prohibiting nighttime riding is not universal. In addition, an increasing number of states and counties are opening roadways to recreational ORV use and a daylight restriction is often not included (Weintraub and Best 2021). Safety experts, including the Consumer Federation of America, recommend a ban on nighttime riding, including on the road (Weintraub and Best 2021; GAO 2021).

### Limitations

These studies have the limitations inherent in retrospective research and those experienced by other ATV injury prevention researchers. These include incomplete capture of crash and injury records and/or incomplete variable documentation including time of crash and light conditions. In addition, data sources used in this study are more likely to record moderate to severe crashes and injuries, rather than crashes resulting in injuries not needing medical attention or only requiring medical care in an outpatient setting. Moreover, because of limitations in available information, some side-by-side vehicles may have been documented as ATVs and included in the study. However, we hypothesize that this would not significantly bias results, as identified side-by-sides only comprised 9% (367/4292) of cases in our database. Despite these limitations, many of our findings are similar to other reports related to nighttime versus daytime crashes and they build on the highly limited information currently available for this topic.

## Conclusions

Nighttime crash victims, particularly adults, were characterized by more frequent risky behaviors like carrying passengers, alcohol use, and lack of helmets. Whereas the frequency of risky behaviors among youth was largely similar for daytime and nighttime crashes, these behaviors put children at potential risk for injury. Targeted injury prevention strategies are needed. These would include improvements in vehicle design, education about the dangers of nighttime operation, and passage/enforcement of ATV safety laws. Laws with particular relevance to our study would prohibit nighttime riding.

## Data Availability

Data and materials are available to other parties for research purposes after a data sharing agreement plan is agreed upon and signed.

## Abbreviations

ATV: All-terrain vehicle
CPSC: Consumer Product Safety Commission
DNR: Department of Natural Resources
DOT: Department of Transportation
ED: Emergency department
GCS: Glasgow Coma Scale
ICD-9/ICD-10: International Classification of Diseases version 9 and 10
IRB: Institutional Review Board
ISS: Injury Severity Score
MV: Motor vehicle
ORV: Off-road vehicle
ROPS: Rollover protective structure
ROV: Recreational off-highway vehicle
STR: State Trauma Registry
SxS: Side-by-side
UTV: Utility task vehicle
VIN: Vehicle identification number

## References

[CR1] Aitken ME, Graham CJ, Killingsworth JB, Mullins SH, Parnell DN, Dick RM (2004). All-terrain vehicle injury in children: Strategies for prevention. Inj Prev.

[CR2] Boyd CR, Tolson MA, Copes WS. Evaluating trauma care: the TRISS method. Trauma Score and the Injury Severity Score. J Trauma. 1987;27:370–8. 3106646

[CR3] Campbell BT, Kelliher KM, Borrup K, Corsi J, Saleheen H, Bourque MD, Lapidus G (2010). All-terrain vehicle riding among youth: How do they fair?. J Pediatr Surg.

[CR4] Consumer Federation of America. All-terrain vehicle (ATV) safety crisis: America's children at risk. Link to pdf available at: http://consumerfed.org/wp-content/uploads/2010/08/atv-safety-crisis-2003-final-all.pdf. Accessed January 2, 2021.

[CR5] Denning G, Harland K, Ellis D, Jennissen C (2013). More fatal all-terrain vehicle crashes occur on the roadway than off: increased risk-taking characterises roadway fatalities. Inj Prev.

[CR6] Denning G, Harland K, Jennissen C. Age-based risk factors for pediatric ATV-related fatalities. Pediatrics. 2014;134(6):1094–102. 10.1542/peds.2014-1993 25422012

[CR7] Denning G, Jennissen C (2018). Pediatric and adolescent injury in all-terrain vehicles. Special Issue: Epidemiology of youth injury in adventure and extreme sports. Res Sports Med.

[CR8] Denning G, Jennissen C, Harland K, Ellis D, Buresh C (2013). All-terrain vehicles (ATVs) on the road: A serious traffic safety and public health concern. Traffic Inj Prev.

[CR9] Denning G, Jennissen C, Harland K, Ellis D, Buresh C. Off-highway vehicle parks: Combining environment, knowledge, and enforcement for all-terrain vehicle injury prevention. Accid Anal Prev. 2013b;52:64–70. 10.1016/j.aap.2012.12.015. 10.1016/j.aap.2012.12.015 23298708

[CR10] Denning GM, Jennissen CA (2016). All-terrain vehicle fatalities on paved roads, unpaved roads, and off-road: Evidence for informed roadway safety warnings and legislation. Traffic Inj Prev.

[CR11] GAO. All-terrain vehicles: How they are used, crashes, and sales of adult-sized vehicles for children's use. 2010 Report to congressional committees (GAO-10-418). Available at: http://www.gao.gov/new.items/d10418.pdf. Accessed January 2, 2021.

[CR12] Hafner JW, Hough SM, Getz MA, Whitehurst Y, Pearl RH (2010). All-terrain vehicle safety and use patterns in central Illinois youth. J Rural Health.

[CR13] Helmkamp JC, Aitken ME, Lawrence BA (2009). ATV and bicycle deaths and associated costs in the United States, 2000-2005. Public Health Rep.

[CR14] Jennissen C, Castaneda C, Long A, Denning G. From the American Academy of Pediatrics Council on Injury, Violence, and Poison Prevention Program. Youth-size ATV seat design: Variability and lack of consistent changes in vehicles designated for different ages demonstrates need for evidence-based standardization. Pediatrics. 2018;141(1) Meeting Abstract. Available at: http://pediatrics.aappublications.org/content/141/1_MeetingAbstract/82. Accessed 2 Jan 2021.

[CR15] Jennissen C, Harland K, Denning G. Characteristics of side-by-side vehicle crashes and related injuries as determined using newspaper reports from nine U.S. states. Safety. 2016a;2(2):10. Full text pdf available online at http://www.mdpi.com/2313-576X/2/2/10. Accessed 2 Jan 2021. 10.3390/safety2020010 PMC9380433 35979514

[CR16] Jennissen C, Harland K, Wetjen K, Denning G. The effect of passengers on all-terrain vehicle crash mechanisms and injuries. Safety. 2016b;2(1):1. Full text pdfavailable online at https://www.mdpi.com/2313-576X/2/1/1. Accessed 2 Jan 2021.

[CR17] Jennissen C, Miller N, Tang K, Denning G. Optimising seat design for all-terrain vehicle injury prevention: wide variability illustrates need for evidence-based standardisation. Inj Prev. 2014;20(2):88–96. 10.1136/injuryprev-2013-040786. 10.1136/injuryprev-2013-040786 23838558

[CR18] Jennissen C, Reaney M, Denning G (2016). Recreational off-highway vehicle crashes resulting in victims being treated at a regional trauma center. Inj Epidemiol.

[CR19] Lord S, Tator CH, Wells S (2010). Examining Ontario deaths due to all-terrain vehicles, and targets for prevention. Can J Neurol Sci.

[CR20] Lower T, Trotter M (2014). Adoption of quad bike crush prevention devices on Australian dairy farms. J Agromedicine.

[CR21] Mazotas I, Toal M, Borrup K, Saleheen H, Hester AL, Copeland D, Danielson PD, DeRoss A, Lapidus G, Bentley G, Thaker S, Campbell BT (2014). A prospective, multi-institutional study of pediatric all-terrain vehicle crashes. J Trauma Acute Care Surg.

[CR22] Merrigan TL, Wall PL, Smith HL, Janus TJ, Sidwell RA (2011). The burden of unhelmeted and uninsured ATV drivers and passengers. Traffic Inj Prev.

[CR23] Meyers M. All-terrain vehicle safety—Potential effectiveness of the Quadbar as a crush prevention device. Safety. 2016;2(1):3. Full text pdf available online at https://www.mdpi.com/2313-576X/2/1/3/htm. Accessed 2 Jan 2021.

[CR24] Qin ES, Jennissen CA, Wadman CA, Denning GM. Using geospatial mapping to determine the impact of all-terrain vehicle crashes on both rural and urban communities. West J Emerg Med. 2017;18(5):913–22. 10.5811/westjem.2017.6.34404 PMC5576628 28874944

[CR25] Rodgers GB, Adler P (2001). Risk factors for all-terrain vehicle injuries: A national case-control study. Am J Epidemiol.

[CR26] Rostas J, Donnellan K, Gonzalez R, Brevard S, Ahmed N, Rogers E, Stinson J, Porter J, Replogle W, Simmons J (2013). Helmet use is associated with a decrease in intracranial hemorrhage following all-terrain vehicle crashes. J Trauma Acute Care Surg.

[CR27] Rowe B, Milner R, Johnson C, Bota G (1994). The association of alcohol and night driving with fatal snowmobile trauma: a case-control study. Ann Emerg Med.

[CR28] Sanfilippo JA, Winegar CD, Harrop JS, Albert TJ, Vaccaro AR (2008). All-terrain vehicles and associated spinal injuries. Spine.

[CR29] Specialty Vehicle Institute of America (SVIA). State all-terrain vehicle requirements. (Feb 2018). Available at: https://svia.org/wp-content/uploads/2017/11/Summary_Chart_February_2016.pdf. Accessed January 2, 2021.

[CR30] Topping, J. 2018 Annual report of ATV-related deaths and injuries. (Feb 2020). Available online at: https://www.cpsc.gov/s3fs-public/2018AnnualReportofATVRelatedDeathsandInjuries.pdf?VGaf1cuZ_D0SGxct2eRpZUwcgME4LKDy. Accessed on January 2, 2021.

[CR31] Touma BJ, Ramadan HH, Bringman JJ, Rodman S (1999). Maxillofacial injuries caused by all-terrain vehicle accidents. Otolaryngol Head Neck Surg.

[CR32] Weintraub R, Best M. ATVs on roadways: a safety crisis. Report by the Consumer Federation of America. http://www.consumerfed.org/pdfs/ATVs-on-roadways-03-2014.pdf. Accessed 2 Jan 2021.

[CR33] Williams A, Oesch S, McCartt A, Teoh E, Sims L (2014). On-road all-terrain vehicle (ATV) fatalities in the United States. J Saf Res.

